# The association among cancer-antigen-125 and pre-eclampsia and estimated fetal weight: A case-control study

**DOI:** 10.18502/ijrm.v22i8.17238

**Published:** 2024-10-14

**Authors:** Hamideh Gholami, Lida Garrosi, Shabnam Tofighi, Zahra Ghadimi

**Affiliations:** ^1^Department of Oncology, Mousavi Hospital, Zanjan University of Medical Sciences, Zanjan, Iran.; ^2^Department of Obstetrics and Gynecology, Mousavi Hospital, Zanjan University of Medical Sciences, Zanjan, Iran.; ^3^Department of Obstetrics and Gynecology, Zanjan University of Medical Sciences, Zanjan, Iran.

**Keywords:** CA-125, Pre-eclampsia, Birth weight.

## Abstract

**Background:**

Although an optimal screening model is still under investigation, pre-eclampsia is a leading cause of perinatal morbidity. The association between cancer-antigen-125 (CA-125) and pre-eclampsia has been discussed in several studies recently.

**Objective:**

We aimed to determine the association between CA-125 and preeclampsia and estimated fetal weight.

**Materials and Methods:**

This case-control study was performed on 30 pregnant women with pre-eclampsia who were homogenized in terms of age, body mass index, and gestational age with 30 normal pregnant women. Participants were recruited via convenience sampling. The level of CA-125 in blood at the time of termination of pregnancy was measured by Enzyme Linked Immunosorbent Assay. The urine sample was used to check proteinuria. Blood pressure and pregnancy outcomes were assessed and recorded.

**Results:**

The mean serum CA-125 level in study group was considerably higher than control group (p < 0.001). Elevating the level of CA-125 has increased the likelihood of pre-eclampsia by 1.5 times. A significant direct correlation was obtained between CA-125 level and the amount of urinary protein (r = 0.605, p < 0.001). Also, a significant but negative correlation was obtained between the CA-125 level and the estimated weight of the fetus (r = -0.593, p < 0.001).

**Conclusion:**

Increasing the serum level of CA-125 with high sensitivity and specificity is significantly associated with the occurrence of pre-eclampsia and estimated fetal weight.

## 1. Introduction

Pre-eclampsia impacts both mothers and their infants significantly. Effective prenatal care facilitates early detection and management through timely delivery, which is crucial in lowering maternal mortality. Despite this, maternal morbidity remains high, with pre-eclampsia being a major reason for pregnant women's admission to intensive care units in developed nations (1, 2). Additionally, pre-eclampsia contributes significantly to fetal mortality and morbidity due to its adverse effects on the fetus and associated prematurity (3). In developing countries, where limited prenatal care hampers effective monitoring of pre-eclampsia, maternal mortality rates are notably high, with approximately 50,000 deaths annually (4).

The cancer-antigen-125 (CA-125) marker is a biomarker, which is abnormally expressed on the surface of epithelial cells (5, 6). CA-125 antigen is present in approximately 80% of ovarian epithelial carcinomas and is also found in various nonmalignant pelvic conditions, including endometriosis, fibroids, pregnancy, pelvic inflammation, and liver disease (7, 8). While fetal chorion, amniotic fluid, and maternal decidua are known to contribute to elevated serum CA-125 levels during the first trimester of pregnancy and the postpartum period, the exact patterns of CA-125 fluctuations throughout the perinatal period remain unclear (9). In cases of pre-eclampsia, it appears that inadequate trophoblastic invasion and the subsequent inflammatory responses in the placenta lead to increased production of biomarkers such as CA-125 (10). Consequently, CA-125 can serve as an indicator of the intensity of inflammation in pre-eclampsia and may be a useful supplementary test for distinguishing between atypical or severe pre-eclampsia and less severe forms (11).

Previous research has indicated that CA-125 levels rise during pregnancy. However, more recent studies have found that this marker's levels remain relatively stable throughout a normal pregnancy (12–14). CA-125 levels increase during the first trimester but are lower compared to levels observed in ovarian cancer. The marker does not show significant changes in the second and third trimesters (15, 16). CA-125 is undetectable before pregnancy but increases following ovulation. Therefore, in women who are pregnant and exhibit elevated tumor marker levels, pregnancy should be considered as a possible cause (17).

Recent studies have shown an increase in serum CA-125 levels in participants with pre-eclampsia compared to normal pregnancies (1, 18, 19). The mechanism of increase in CA-125 level in pre-eclampsia is the isolation and destruction of decidua and the separation of trophoblasts from decidua (20).

The role of CA-125 in the prenatal period is still under study, and several studies have been performed on the association of this tumor marker with pregnancy and its consequences, such as abortion, ectopic pregnancy, and even preeclampsia. Given the significance of pre-eclampsia and its associated risks for both maternal and fetal health, coupled with the limited clinical research on the correlation between CA-125 serum levels and hypertensive disorders in pregnancy, along with the inconsistent findings reported in existing studies, and the importance of this issue led us to investigate the association between CA-125 and pre-eclampsia and estimated fetal weight (EFW).

## 2. Materials and Methods

This case-control study was performed on 60 pregnant women (28–40 wk) who referred to Ayatollah Mousavi hospital in Zanjan, Iran from September 2020 to June 2021 and were divided into 2 study groups consisting of women with pre-eclampsia (n = 30) and the control group of non-preeclamptic women (n = 30).

In this study, the diagnostic and classification criteria for pre-eclampsia followed the guidelines established by the American College of Obstetricians and Gynecologists, categorizing it into non-severe types (9). Pregnancy-induced hypertension was defined using 2 blood pressure measurements taken at least 4 hr apart, with systolic values exceeding 140 mmHg but below 160 mmHg, and diastolic values greater than 90 mmHg but less than 110 mmHg. Measurements were obtained using appropriately sized cuffs. To further classify the study group, we employed criteria from the American College of Obstetricians and Gynecologists, which included a 24-hr proteinuria threshold of 300 mg/day or a positive result on a dipstick test. Such participants were eligible to have a sonography report without abnormalities in the ovaries and uterus.

During the registration process, for each sample in the study group, age, body mass index (BMI), and gestational age-matched sample with normal pregnancy were included in the control group, and this process continued until the sample size was completed. Convenience sampling was applied to this study. Exclusion criteria were dissatisfaction with participation in the study, underlying disease (diabetes, cardiovascular disease, liver and kidney disease, chronic hypertension, lupus, rheumatoid arthritis, cancer), any uterine and ovarian lesions (benign or malignant), and history of smoking, chorioamnionitis, colitis, cirrhosis, tuberculosis, pelvic inflammation, or endometriosis.

The concentration of CA-125 at the time of termination of pregnancy was measured by the ELISA method with a canage kit (Mehr Azmoon Co., Iran). In all cases a blood sample of 5–10 cc for measuring the serum CA-125 level was collected in tubes without anticoagulants and delivered to the hospital laboratory.

In order to check the proteinuria, the participants' urine samples after centrifugation and analysis with sulfasalic acid were checked macroscopically and microscopically. Participants' blood pressure was measured and recorded twice by a single operator with a suitable and standard sphygmomanometer. In participants with pre-eclampsia, fetal growth and EFW (EFW) were assessed routinely before pregnancy termination in 37 wk and 6 days. In the control group, at the same gestational age as in the study group, fetal growth was assessed by ultrasound, and quadruple biometrics were performed before pregnancy termination at the expense of the researcher. The sonographic assessment was conducted with a single imaging device, specifically a real-time grayscale scanner equipped with a 3.5 MHz transducer. A linear array probe was used preferentially for the examination. EFW was calculated using Shepard's formula. EFW in each case was checked and proved by 2 radiologists. Birth weight was also measured and recorded in both groups by a single calibrated scale with an accuracy of 0.001 gr by 2 researchers at the earliest opportunity after birth.

### Sample size

According to previous research by Ozat et al. (21), the maximum sample size was estimated to be 30 people for each group. The sample size was calculated using the formula of comparing 2 averages of type one error (0.01), 90% power, d (6.4), δ (5), and 20 people were calculated in each group, which was due to an increase in the power and accuracy of the study, we increased the sample size of each group to 30 people. 


$$n=\frac{2{\left(z_{1-\propto /2}{+z}_{1-\beta }\right)}^{2}\delta^{2}}{d^{2}}$$

All pregnant women with a gestational age of 28–40 wk who were diagnosed with pre-eclampsia (an increase in blood pressure above 140/90 mmHg on 2 occasions with an interval of 4 hr, or one-time blood pressure of 160/110 mmHg, as well as proteinuria above 300 mg per day or a positive stick dip test and impaired liver function tests and a decrease in platelets after 20 wk of their pregnancy) was recruited in the study. If they had an ultrasound scan showing no abnormality in the ovary and uterus and informed consent, they were included in the study as a study group. For each sample in the study group, one sample of a mother with a normal pregnancy and gestational age equivalent to the study group was included in the control group, and this process continued until the sample volume was completed.

### Ethical considerations

Ethical approval was obtained from the Ethics Committee of the Zanjan University of Medical Sciences, Zanjan, Iran (Code: IR.ZUMS.REC.1399.116). All enrolled participants provided informed written consent.

### Statistical analysis

For the statistical analysis, the statistical software SPSS version 23.0 for windows (IBM, Armonk, New York) was used. Mean and standard deviation were used to describe quantitative variables, and frequency (percentage) was used to describe qualitative variables. Chi-square test was used to compare qualitative variables between 2 groups, and *t* test or its non-parametric equivalent (Mann-Whitney test) was used to compare quantitative variables. To investigate the diagnostic value of CA-125 in predicting pre-eclampsia, receiver operating characteristic (ROC) curve analysis was used, and the best cut point for CA-125 along with its sensitivity and specificity for predicting pre-eclampsia was calculated. Correlation between CA-125 and other study parameters were checked through Pearson or Spearman correlation test. A multivariate logistic regression model was used to predict the relationship between pre-eclampsia and background factors.

## 3. Results

In this study, the groups were completely matched in terms of age, BMI, and gestational age. The study groups were also comparable in the number of gravida, parity, and history of abortion. The mean age of participants in study group was 29.47 ± 5.90, and in control group was 28.17 ± 4.90 (Table I).

**Figure 1:**
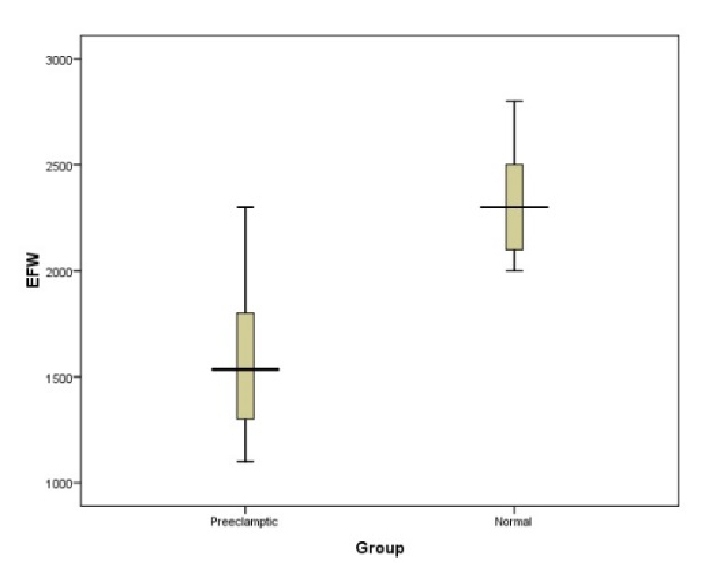
Comparison of estimated fetal weight (EFW) in pre-eclamptic and normal women.

In the present study, a mild significant direct correlation was obtained between the level of CA-125 and the amount of urinary protein (correlation coefficient equal to 0.605, p < 0.001). Also, a significant but negative correlation was obtained between the level of CA-125 and the estimated weight of the fetus (correlation coefficient equal to -0.593, p < 0.001) (Figure 1). However as shown in table II, no significant association was observed between CA-125 level and other indicators such as maternal age, maternal BMI, and weight birth.

In terms of fetal condition, the mean EFW based on ultrasound in 2 groups with and without pre-eclampsia was 1618.40 ± 64.51 gr and 2315.00 ± 39.47 gr, respectively, which was significantly lower in the group with pre-eclampsia (p < 0.001). Moreover, the mean birth weight of the infants in the 2 groups with and without pre-eclampsia was 1888.04 ± 300.93 gr and 1764.80 ± 356.34 gr, respectively, which were significantly different (p < 0.001) (Table I).

In this study EFW, birth weight and platelet level in mother's blood showed an indirect significant correlation with CA-125 level (p < 0.001). In addition, systolic blood pressure, diastolic blood pressure, urine protein, aspartate aminotransferase, alanine transaminase, alkaline phosphatase, and lactate dehydrogenase showed a direct significant correlation with CA-125 level (p < 0.001). However as shown in table II, no significant association was observed between CA-125 level and other indicators such as maternal age, maternal BMI, and birth weight (Table II).

In the present study, a mild significant direct correlation was obtained between the level of CA-125 and the amount of urinary protein (correlation coefficient equal to 0.605, p < 0.001). Also, a significant but negative correlation was obtained between the level of CA-125 and the EFW (correlation coefficient equal to -0.593, p < 0.001) (Figure 1).

**Figure 2:**
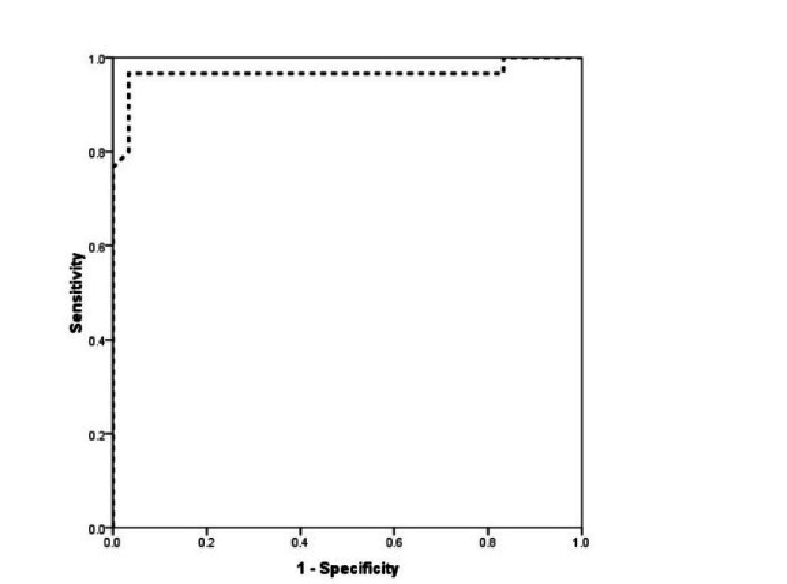
Rock curve, value of increasing CA-125 in predicting the occurrence of pre-eclampsia.

The mean serum CA-125 level in the 2 groups with and without pre-eclampsia was 36.65 ± 11.02 units/ml and 15.43 ± 5.27 units/ml, respectively, which was considerably higher in the group with pre-eclampsia (p < 0.001). Based on the analysis of the area under the ROC curve, the ROC evaluation of CA-125 level was strongly able to predict the occurrence of pre-eclampsia (ROC = 0.966, 95% CI: 0.911–1.000). Based on this, the best CA-125 cut-off point for predicting pre-eclampsia was determined to be 25, which was able to predict pre-eclampsia with a sensitivity of 96.7% and a specificity of 95.9%. Based on the multivariate logistic regression model and with the presence of background factors including mother's age, mother's BMI, parity, and history of abortion, an increase in CA-125 level, increased the chance of pre-eclampsia by 1.5 times (OR = 1.44, 95% CI: 1.18–1.7, p = 0.001) (Figure 2).

**Table 1:** Baseline characteristics of participants


**Item**		**With pre-eclampsia**	**Without pre-eclampsia**	**P-value**
Age (yr)*		29.47 ± 5.90	28.17 ± 4.90	0.60
Gravid status**				
	**1**	8 (26.7)	8 (26.7)	
	**2**	12 (40.0)	12 (40.0)	
	**3**	8 (26.7)	8 (26.7)	
	**4**	1 (3.3)	1 (3.3)	
	**5**	1 (3.3)	1 (3.3)	1.00
Parity status**				
	**0**	9 (30.0)	12 (40.0)		
	**1**	15 (50.0)	12 (40.0)		
	**2**	5 (16.7)	6 (20.0)		
	**3**	1 (3.3)	0 (0.0)	0.60
Live birth**				
	**0**	9 (30.0)	12 (40.0)		
	**1**	15 (50.0)	12 (40.0)		
	**2**	6 (20.0)	6 (20.0)	0.68
History of abortion**				
	**0**	24 (80.0)	24 (80.0)		
	**1**	6 (20.0)	5 (16.7)	
	**2**	0 90.0)	1 (3.3)	0.58
Mean EFW (gr)***		1618.40 ± 64.51	2315.00 ± 39.47		< 0.001
Mean birth weight (gr)***		1700.80 ± 220.48	2389.17 ± 65.48		< 0.001
*Data presented as Mean ± SD, Chi-square test. **Data presented as n (%), *t* test. ***Data presented as Mean ± SD, *t* test. EFW: Estimated fetal weight				

**Table 2:** The correlation between CA-125 level and other parameters


**Item**	**Correlation coefficient**	P-value*
**Mother age**	0.024	0.85
**BMI**	0.132	0.31
**EFW**	-0.593	< 0.001
**Birth weight**	-0.110	0.40
**Systolic blood pressure**	0.508	< 0.001
**Diastolic blood pressure**	0.638	< 0.001
**Urine protein level**	0.605	< 0.001
**AST level**	0.641	< 0.001
**ALT level**	0.624	< 0.001
**ALKP**	0.522	< 0.001
**LDH**	0.545	< 0.001
**Platelet**	-0.698	< 0.001
BMI: Body mass index, EFW: Estimated fetal weight, AST: Aspartate aminotransferase, ALT: Alanine transaminase, ALKP: Alkaline phosphatase, LDH: Lactate dehydrogenase, *Pearson correlation coefficient		

## 4. Discussion

This study shows a significant association between CA-125 level in maternal serum with pre-eclampsia and EFW and birth weight. There is evidence of an increase in this marker in cases of pre-eclampsia and eclampsia. In some cases, an increase in serum CA-125 levels has been associated with adverse pregnancy outcomes. In the present study we determined the value of CA-125 in predicting the occurrence of pre-eclampsia and its correlation with maternal and neonatal characteristics. The dominant finding in our study was that, firstly, the serum level of the CA-125 marker in women with pre-eclampsia was much higher than in those without pre-eclampsia. Therefore, the increase in serum levels of this marker increased the risk of this event by 1.5 times.

In order to determine the value of CA-125 in predicting pre-eclampsia, as a second finding, we found that there was a direct correlation between CA-125 and the rate of proteinuria, as well as an inverse correlation between CA-125 and the EFW. This issue has 2 important points. First, an increase in CA-125 levels can also predict the severity of pre-eclampsia because the severity of pre-eclampsia is also known based on the severity of proteinuria. Second, the CA-125 assessment alone can predict the consequences associated with pre-eclampsia, including birth weight, EFW, and, indeed, fetal intrauterine growth restriction (IUGR). Therefore, in this study, the predictive value of increased CA-125 levels (especially > 25 units/ml) in the prediction of pre-eclampsia, as well as its severity, was fully established.

A review study highlighted a significant difference in CA-125 levels between participants with and without pre-eclampsia (22). Similarly, in another study researchers found a significant correlation between CA-125 levels and proteinuria, platelet count, and both systolic and diastolic blood pressure (21).

In our study, the optimal CA-125 cut-off point for predicting pre-eclampsia was identified as 25. In another study, a slightly higher cut-off of 26.85, with a sensitivity of 87.7% and a specificity of 93.3% has been reported (18), which is close to our finding. Conversely, another study established a cut-off of 50 units/ml, showing a sensitivity of 93.7% and a specificity of 88.0% for predicting pre-eclampsia (21), which differs significantly from our cut-off. This higher cut-off point in their study resulted in lower sensitivity for predicting pre-eclampsia. These variations in cut-off points may arise from differences in study design, patient selection criteria, CA-125 measurement methods, and demographic factors.

What is of interest today is the exploration of the pathophysiological basis of the association between elevated CA-125 levels and the development of pre-eclampsia (23). However, some believe that the effect of CA-125 directly on the vascular receptors provides the basis for the occurrence of pre-eclampsia. Recent studies have shown that increasing the level of this marker is not a direct factor in increasing the risk of pre-eclampsia, but rather increase the affinity of MUC16 to some immunological cells, such as natural killer cells and monocytes, can involve the pathological process leading to hypertension and proteinuria and eventually pre-eclampsia (24, 18).

Schröcksnadel et al. were pioneers in comparing plasma CA-125 levels among 3 groups: 50 healthy non-pregnant women, 50 women with hypertensive disorders during pregnancy, and 50 healthy women with singleton pregnancies at term. Their study found no significant differences in CA-125 levels between these groups (25). Similarly, in a study researchers reported no significant differences in CA-125 concentrations related to pregnancy outcome or trimester (23). They observed that maternal serum CA-125 levels are elevated during the first and third trimesters of pregnancy but did not find an association with pre-eclampsia (19). These studies are in contrast with the results of our study. The various methods and the difference between the gestational ages of participants are the most probable reasons.

A recent study reported significant difference among 3 groups regarding to CA-125 value in maternal serum and it was elevated significantly in mild and severe pre-eclampsia, it correlated with the severity of preeclampsia (26) which in some terms is along with our results.

In this study, there was a significant and direct correlation between serum CA-125 level and systolic blood pressure, diastolic blood pressure, urine protein level, aspartate aminotransferase level, ALT level, ALKP, and LDH. In direct correlation with platelets, these relationships were found in the results of some previous studies. The study demonstrated a significant direct correlation between mean CA-125 levels and both systolic and diastolic blood pressure (18). In contrast, in a study, researchers found associations with systolic and diastolic blood pressure, as well as platelet count, uric acid, and urine protein concentration (21). Also, a significant direct correlation between CA-125 levels and systolic and diastolic blood pressure, platelet count, uric acid, and urine protein concentration is reported in another study (26).

In the current study, only IUGR and the use of blood pressure medication in the pre-eclampsia group had a significant impact on CA-125 levels. Additionally, a comparison based on the presence of IUGR indicated significant differences in mean values for systolic and diastolic blood pressure, platelet count, creatinine, uric acid, CA-125, hemoglobin, and birth weight. The study found a notable inverse correlation between mean CA-125 levels and both platelet count and birth weight. Similar inverse relationships with birth weight and fetal weight were observed in other studies (14, 21).

In this study, a significant difference was observed between EFW by sonography and birth weight. All other studies have approved this finding (16, 21, 27). The reason for this common finding is that according to the guidelines, in patients with severe pre-eclampsia, we have to terminate the pregnancy earlier than 36 wk and delivery is performed at a lower gestational age in comparison with normal pregnancies.

The advantages of the present study include focusing on the occurrence of pre-eclampsia and matching some demographic variables in the 2 groups (age, BMI, and gestational age). However, the limited sample size may have influenced the results and the assessment of relationships, potentially impacting the identification of significant associations.

## 5. Conclusion

In conclusion, increasing the serum level of CA-125 (especially > 25 units per mg) with high sensitivity and specificity can predict the occurrence of pre-eclampsia. Elevated CA-125 levels are directly related to proteinuria severity and inversely related to fetal estimated weight. Due to the relationship between this marker and a wide range of other variables (systolic blood pressure, diastolic blood pressure, urine protein level, aspartate aminotransferase level, ALT level, ALKP, LDH, and platelet), paying attention to its serum level along with other para-clinical variables can help in better diagnosis and management of patients. New clinical methods with different approaches and the integration and combination of clinical knowledge and experience will play an important role in generalizing the results of evidence-based clinical studies regarding the treatment and survival of patients.

##  Data availability

Data supporting the findings of this study are available upon reasonable request from the corresponding author.

##  Author contributions

H. Gholami and Z. Gadimi designed the study and conducted the research. L. Garrosi and Sh. Tofighi monitored, evaluated, and analyzed the result of the study and reviewed the article. All authors approved the final manuscript and take responsibility for the integrity of the data.

##  Conflict of Interest

The authors declare no conflict of interest.
